# Activation of IFN-γ/STAT/IRF-1 in Hepatic Responses to *Klebsiella pneumoniae* Infection

**DOI:** 10.1371/journal.pone.0079961

**Published:** 2013-11-06

**Authors:** Yi-Chun Lin, Min-Chi Lu, Chingju Lin, Ming-Ko Chiang, Ming-Shiou Jan, Hui-Ling Tang, Hsu-Chung Liu, Wea-Lung Lin, Chih-Yang Huang, Chuan-Mu Chen, Yi-Chyi Lai

**Affiliations:** 1 Department of Life Sciences, Agricultural Biotechnology Center, National Chung Hsing University, Taichung, Taiwan; 2 Division of Infectious Diseases, Department of Internal Medicine, Chung Shan Medical University Hospital, Taichung, Taiwan; 3 Department of Microbiology and Immunology, Chung Shan Medical University, Taichung, Taiwan; 4 Institute of Microbiology and Immunology, Chung Shan Medical University, Taichung, Taiwan; 5 Department of Physiology, School of Medicine, China Medical University, Taichung, Taiwan; 6 Department of Life Science, National Chung Cheng University, Chia-Yi, Taiwan; 7 Department of Veterinary Medicine, National Chung Hsing University, Taichung, Taiwan; 8 Division of Gastroenterology and Hepatology, Department of Internal Medicine, China Medical University Hospital, Taichung, Taiwan; 9 School of Medicine, Chung Shan Medical University, Taichung, Taiwan; 10 Division of Chest Medicine, Department of Internal Medicine, Chung Shan Medical University Hospital, Taichung, Taiwan; 11 Department of Pathology, Chung Shan Medical University Hospital, Taichung, Taiwan; 12 Graduate Institute of Chinese Medical Science, China Medical University, Taichung, Taiwan; 13 Graduate Institute of Basic Medical Science Chinese Medical Science, China Medical University, Taichung, Taiwan; 14 Department of Health and Nutrition Biotechnology, Asia University, Taichung, Taiwan; Université d'Auvergne Clermont 1, France

## Abstract

**Background:**

*Klebsiella pneumoniae*-caused liver abscess (KLA) has become a health problem in Taiwan and is continually reported in other countries. Diabetes mellitus, the most common metabolic disorder, underlies half of the KLA patients in Taiwan. The clinical impact of KLA has been well-documented. Nevertheless, the molecular basis regarding how *K. pneumoniae* causes liver infection, particularly in diabetic individuals, remains unclear.

**Methodology/Principle Findings:**

Auto-bioluminescence-expressing *K. pneumoniae* was inoculated into diabetic mice and age-match naïve control. With the use of *in* *vivo* imaging system, translocation of the bioluminescence-expressing *K. pneumoniae* from intestine to extraintestinal organs, mainly the liver, was noted in 80% of the diabetic mice, whereas the same bacteria causes extraintestinal infections in only 31% of naïve mice. Besides increased morbidity, the severity of hepatic tissue injury was also enhanced in the *K. pneumoniae*-infected diabetic mice. Upon *K. pneumoniae* infection, IFN-γ production was significantly evoked in the liver. To mediate IFN-γ signal, STAT (signal transducers and activators of transcription) 1 and 3 were activated in hepatocytes, and so was the expression of IRF (interferon regulatory factor)-1. Moreover, accumulation of neutrophils which was triggered by prolonged production of IL-1β and MIP-2, and significant increases in the level of active caspase 3 and phospho-eIF2α, were exclusively revealed in the *K. pneumoniae*-infected diabetic mice.

**Conclusion:**

The activation of IFN-γ/STAT/IRF-1 signaling demonstrated by this work emphasizes the role of IFN-γ for mediating the hepatic response to *K. pneumoniae* infection.

## Introduction

*Klebsiella pneumoniae*, a member of the family *Enterobacteriaceae*, frequently involves a wide range of clinical illnesses, such as pneumonia, suppurative infections, meningitis, bacteremia and septicemia. Without immediate treatment, infections caused by this bacterium have poor prognosis with high mortality rates [1]. During 1990s, *K. pneumoniae* surpassed *Escherichia coli* as the primary pathogen for community-acquired pyogenic liver abscess in Taiwan [2]. Since its first recognition, *K. pneumoniae*-caused liver abscess (KLA) is now considered an emerging disease worldwide [3-6]. Distinct from *Escherichia coli*-caused liver abscess, KLA is generically cryptogenic and is frequently complicated in up to 20% of cases with septic metastatic lesions to other organs [7-9]. Meningitis and endophthalmitis are complications that may lead to neurological sequelae [10]. Nevertheless, the cellular mechanism of KLA pathogenesis still awaits elucidation. 

Gamma interferon (IFN-γ) is a proinflammatory cytokine produced by natural killer (NK) cell, NKT, T_H_1 and CD8^+^ T cells that activate macrophages and the related antimicrobial properties. IFN-γ is required for T-cell-mediated immunity against various pathogens, such as *Coxiella burnetii* [11], *Legionella pneumophila* [12], *Listeria monocytogenes* [13], *Staphylococcus aureus* [14], and *Chlamydia pneumoniae* [15]. Besides, the linkage of IFN-γ to the pathogenesis of liver diseases has been reported by several studies [16-18]. Recognition of IFN-γ by IFN-γ receptor (IFNGR1/2) activates the Janus kinase/signal transducers and activators of transcription (JAK/STAT) pathway [19]. STAT1 is the main STAT protein expressed in response to IFN-γ signaling [20]. Through the action of SRC and JAK kinases, IFN-γ also activates STAT3. Phosphorylated STAT1 and STAT3 formed homo- or hetero-dimers, translocated into the nuclei, drove the expression of IFN-γ responsive genes, including IFN regulatory factor-1 (IRF-1) [21]. Subsequent expression of the IFN-γ responsive genes that are related to apoptosis and cell cycle arrest is controlled by IRF-1. IFN-γ induces apoptosis of various cell types, including hepatocytes; however, its mechanism is divergent and involves multiple downstream pathways [22,23]. Generation of reactive oxygen species (ROS) and endoplasmic reticulum (ER) stress were also demonstrated to promote apoptosis of cultured hepatocytes [24]. 

Diabetes mellitus is a metabolic disorder characterized by the loss of glucose homeostasis. Type 1 diabetes is caused by autoimmune-triggered destruction of insulin-producing β cells of the pancreas. Type 2 diabetes is characterized by high blood glucose within the context of insulin-resistance and relative insulin deficiency. Based on the report by World Health Organization, the prevalence of diabetes for all age-groups worldwide was estimated to be 4.4% in 2030. Diabetes underlies half of the KLA patients in Taiwan and increases the incidence of KLA-related septic metastatic lesions [25,26]. Various factors may affect the vulnerability of diabetic individuals to infection, including genetic susceptibility, altered cellular and humoral immune defense mechanisms, poor blood supply, nerve damage, and alterations in metabolism [27]. Nevertheless, the molecular basis regarding how *K. pneumoniae* causes liver infections, particularly in diabetic individuals, still remains unclear. Considering the involvement of IFN-γ in the host response to *K. pneumoniae* has only been demonstrated in a pneumonia model [28], we aimed in this study to investigate the role of IFN-γ/STAT/IRF-1 signaling in hepatic responses to *K. pneumoniae*-caused liver infection. Auto-bioluminescence-expressing *K. pneumoniae* was generated for *in vivo* monitoring dissemination of *K. pneumoniae* from the intestine to the liver. The *K. pneumoniae*-evoked hepatic responses, including the release of inflammatory cytokines, histopathological change of hepatic tissue, activation of IFN-γ/STAT/IRF-1 signaling, apoptosis, and ER stress was analyzed comparatively in the diabetic mice and age-matched control mice.

## Results

### *In vivo* imaging dissemination of auto-bioluminescence-expressing *K. pneumonia*

**Figure 1:**
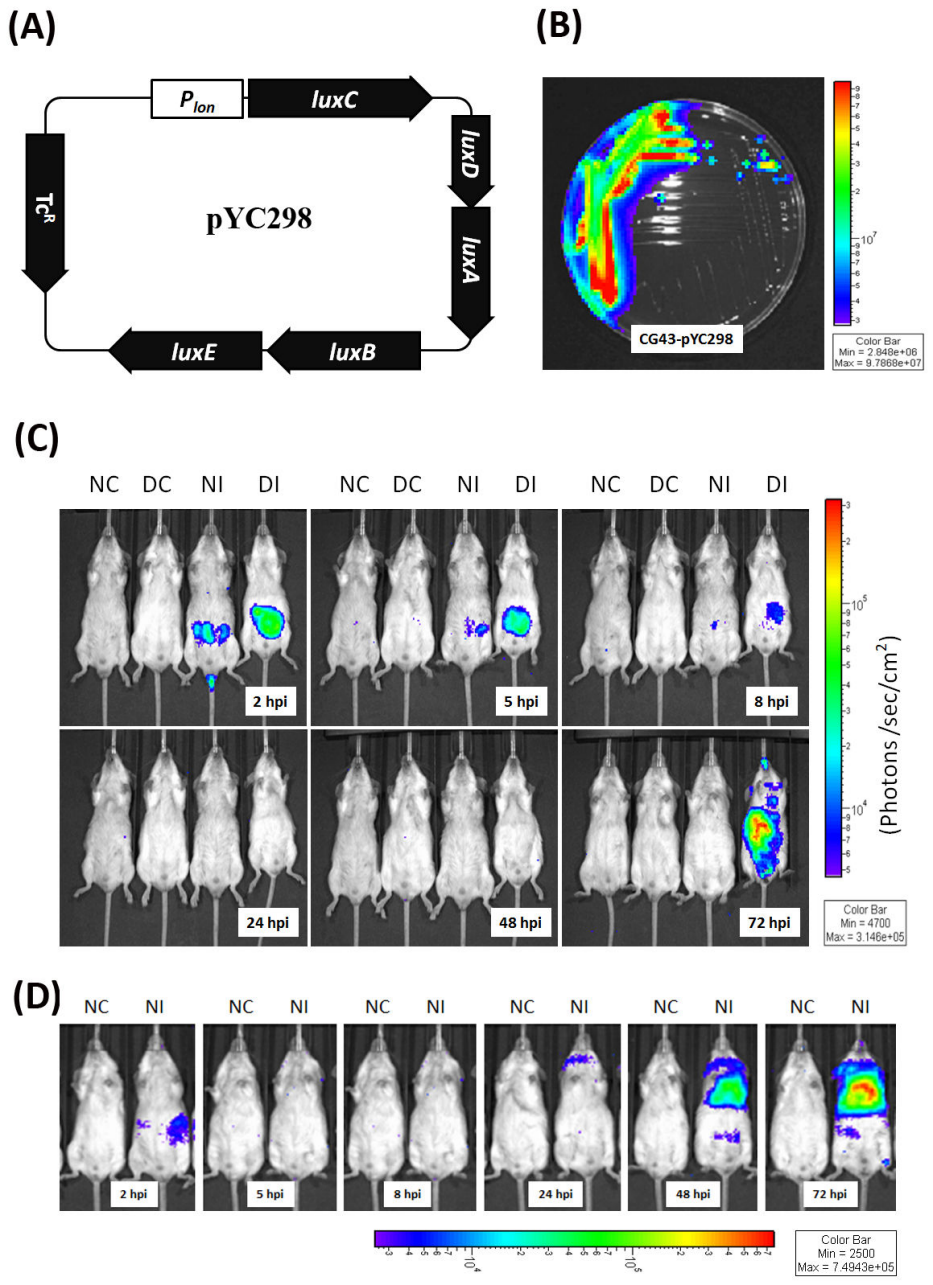
*In* *vivo* imaging of auto-bioluminescence-expressing *K. pneumoniae*. (A) Map of pYC298. (B) Detection of bioluminescence light signals of *K. pneumoniae* CG43-pYC298 cultured on LB plate. (C-D) Groups of 15-wk-old male BALB/c mice were orally inoculated with PBS or 3 × 10^8^ CFU of log-phased *K. pneumoniae* CG43*-*pYC298. The mice were imaged at 2, 5, 8, 24, 48 and 72 hpi using the Xenogen IVIS system. The color overlay on the image represents the photons/second emitted from the mice in accord with the pseudo-color scale shown near to the images. Red represents the highest photons/second, while purple represents the lowest photons/second. The data are expressed as photons/seconds/cm^2^ in PBS-control naïve mice (NC), PBS-control diabetic mice (DC), *K. pneumoniae*-infected naive mice (NI) and *K. pneumoniae*-infected diabetic mice (DI), respectively from left to right.

To monitor *K. pneumoniae* infection comparatively in diabetic and naïve mice, auto-bioluminescence-expressing *K. pneumoniae* was generated by transformation with pYC298 (Figure 1A), which carried the *luxCDABE* operon of *Photorhabdus luminescens* [29] driven by the promoter of Lon protease gene. Bioluminescence light signals can be generated by synergistic production of proteins encoded by the *luxCDABE* operon exclusive supplementary substrate additions. The Lon protease is an ATP-dependent serine protease involved in the control of protein quality which is essential for maintaining bacterial physiology. Expression of the Lon protease was under a strong constitutive promoter, which had 100-fold higher activity than the conventional *lac* promoter (unpublished data). Although its expression might be up-regulated upon stressful conditions [30,31], the use of *lon* promoter in pYC298 allowed for constitutive expression of luciferase *in vivo*. With the use of Xenogen IVIS Imaging System, the auto-bioluminescence-expressing *K. pneumoniae* (Figure 1B) was handily detected by a minimum limit of 1×10^4^ CFU/ml in LB culture. Given that intestinal colonization with *K. pneumoniae* is considered the first step of KLA [32,33], suspension of 3×10^8^ CFU of auto-bioluminescent *K. pneumoniae* was inoculated into groups of diabetic and age-matched naïve mice via an oral route. As shown in Figure 1C, bioluminescence signals were detected primarily in the abdomen of *K. pneumoniae*-infected mice (NI and DI) at 2 hpi (hour post-inoculation), and were continually reduced with a more accelerated rate in the naïve group, suggesting that the inoculums of *K. pneumoniae* were mostly shed through the feces. Although the bioluminescence signal was under the limit of detection by the Xenogen IVIS system at 8, 24, and 48 hpi, small amounts of intestinal *K. pneumoniae* were enough to initiate an extraintestinal infection. As shown in Figure 1C at 72 hpi, the location of the strongest bioluminescent intensity spots (as red color) coincided with the approximate location of liver in the *K. pneumoniae*-infected diabetic mice (DI), whereas no signal was detected in the naïve mice that successfully conquered *K. pneumoniae* invasion (NI). However, once *K. pneumoniae* penetrated the intestinal barrier of naïve mice, it also developed severe extraintestinal infections at 72 hpi (Figure 1D; NC vs. NI). 

### Increased susceptibility of diabetic mice to extraintestinal *K. pneumoniae* infection

**Figure 2:**
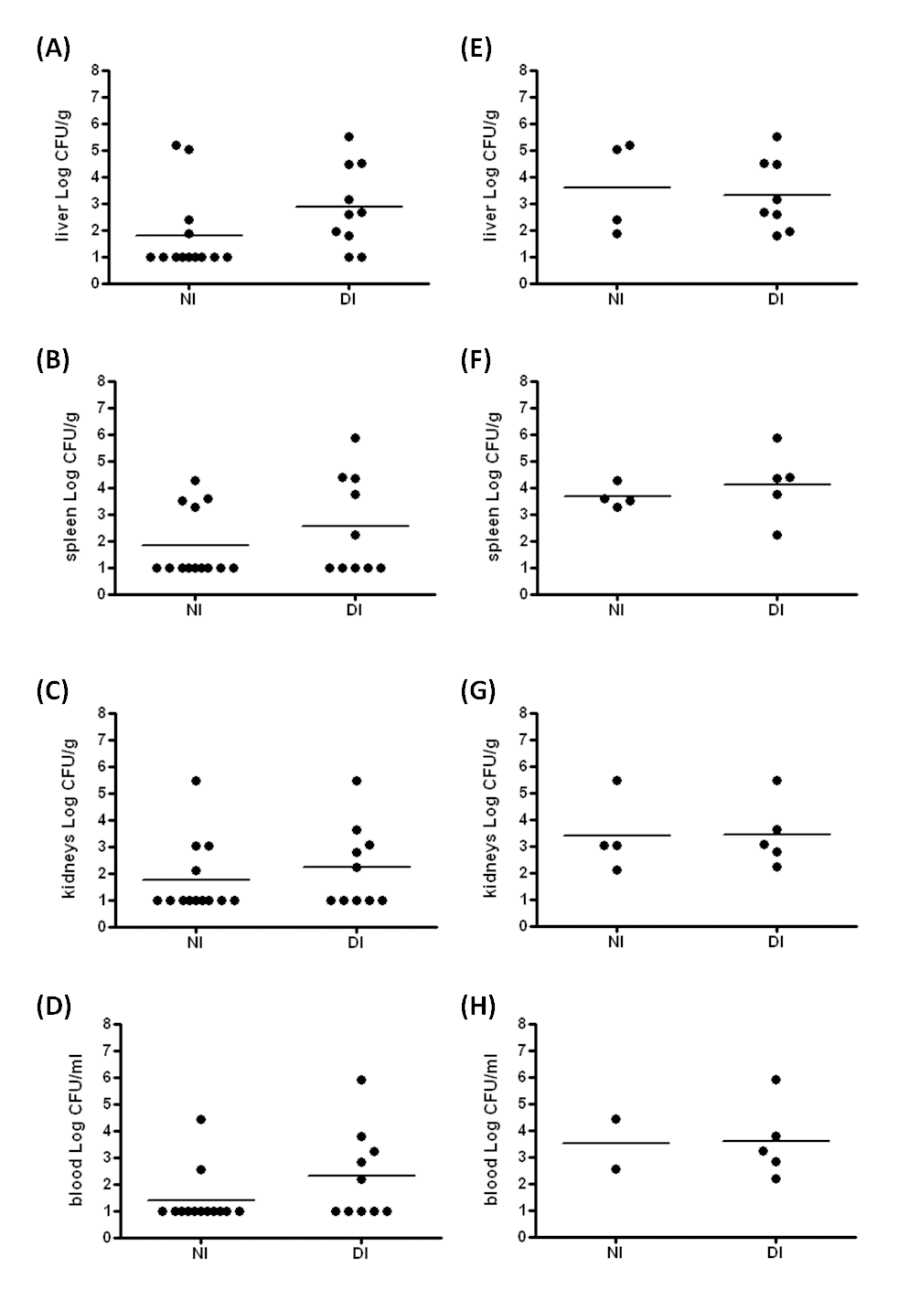
Bacterial burdens of extraintestinal organs upon *K. pneumoniae* infection in diabetic and naïve mice. Liver (A and E), spleen (B and F), kidneys (C and G), and blood (D and H) were retrieved at 72 hpi from the naive mice (NI) and diabetic mice (DI) which were orally inoculated with 3 × 10^8^ CFU of log-phased *K. pneumoniae* CG43*-luxCDABE*. Ten-folded dilutes of the tissue homogenates were plated onto LB-Tc^R^ agar to enumerate CFU. Bacterial burdens were represented as Log CFU/g for tissues and Log CFU/ml for blood. Data from all the *K. pneumoniae*-inoculated naïve and diabetic mice were shown in A to D. Horizontal bars indicate geometric means. The limit of detection was approximately 10 CFU. Samples which yielded no colonies were plotted having the value as 10 CFU g^-1^ tissues. For emphasizing the extraintestinal dissemination of *K. pneumoniae*, samples that yielded no colonies in extraintestinal tissues were considered non-infected. Only data from the *K. pneumoniae*-positive samples were plotted in E to H. Statistical analysis by the Mann-Whitney U test (one-tailed) showed no significant difference between NI and DI groups. The sample sizes in A-D are 13 and 10 in NI and DI groups and are 4 and 8 in E-H for NI and DI groups, respectively.

**Figure 3:**
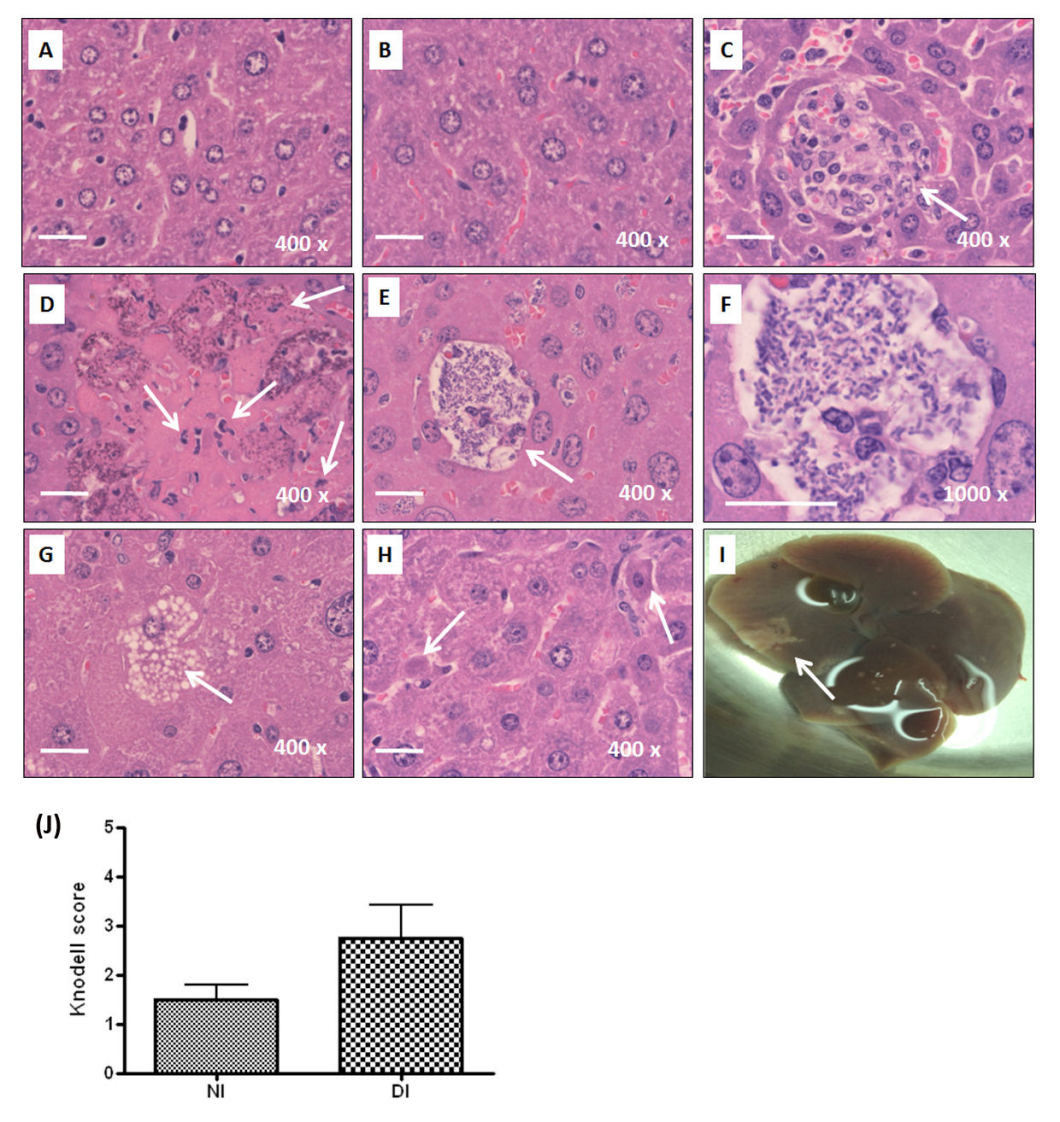
Histopathological examination of liver. For all the experimental groups, liver sections were prepared from the liver retrieved at 72 hpi, stained with H/E, and imaged under microscopic observation with magnification of 400×. Representative liver sections of PBS-control naïve mice (A), PBS-control diabetic mice (B), and the *K. pneumoniae*-infected naïve mice (C) are shown. Infiltrates of neutrophils and lymphocytes are indicated with arrows. Several characteristics revealed on the liver section from the *K. pneumoniae*-infected diabetic mice, including liquefactive necrosis with degeneration of liver parenchyma and inflammatory cells (D), accumulation of *K. pneumoniae* (E and F), ballooning degeneration of hepatocytes (G), and the formation of Councilman body (H) are shown with an indication of arrows. A large hepatic abscess was noted in the right liver lobe of a *K. pneumoniae*-infected diabetic mouse (I). Scale bar represents a distance of 20 μm. (J) Hepatic injury graded by the Knodell necroinflammatory scoring system. Livers were retrieved from *K. pneumoniae*-infected naïve mice (NI; n=4) and diabetic mice (DI; n=8). Statistical analysis by the Mann-Whitney U test (one-tailed) showed no significant difference between NI and DI groups.

The IVIS result suggested that *K. pneumoniae* disseminated into extraintestinal organs more easily in mice with diabetes in comparison with the naïve control. To validate this result, liver, spleen, kidneys, and blood were collected at 72 hpi for bacterial enumeration. Positive culture of *K. pneumoniae* yielded in any of the deep organs indicated the occurrence of an extraintestinal infection. The incidence was therefore defined as the percentage of mice which were infected with *K. pneumoniae* in any of the extraintestinal organs. As shown in Table 1, 80% (8/10) of the *K. pneumoniae*-inoculated diabetic mice developed an extraintestinal infection, whereas the incidence rate was significantly reduced to 31% (4/13) in the naïve group (Table 1; 80% *vs* 31%; *P*=0.036; odds ratio=9; 95% confident interval=1.285-63.025). Among the extraintestinal organs, liver served as the primary site to be invaded by disseminating *K. pneumoniae* from the intestine. Mice with diabetes were more susceptible to develop *K. pneumoniae*-caused liver infection than the naïve mice (Table 1; odds ratio=9). Bacterial burdens in the extraintestinal tissues, including liver, spleen, kidneys, and blood, were determined (Figure 2A-D). To examine hepatic responses to *K. pneumoniae* infections, only mice which had developed liver infections at 72 hpi were included for the subsequent analyses. Of 10 diabetic and 13 naïve mice, 8 and 4 mice respectively were included. As shown in Figure 2E-H, once an infection established, average bacterial burdens of extraintestinal tissues were comparable between the diabetic and naïve groups. Hepatic inflammation and damage was developed in the diabetic and naïve mice that had *K. pneumoniae*-caused liver infection. When compared with the PBS control (Figure 3A and B), infiltrates of granulocytes and lymphocytes were noted at 72 hpi (Figure 3C) and accumulation of neutrophils was enhanced in the diabetic group (Figure 3D). Severe hepatic injuries, characterized as central liquefactive necrosis with degeneration of liver parenchyma and inflammatory cells, were occurred exclusively in the *K. pneumoniae*-infected diabetic mice (Figure 3D). A considerable amount of *K. pneumoniae* were localized within the empty space that was left by cellular necrosis amongst the liver parenchyma (Figure 3E and F). Besides, hepatocytes that underwent ballooning degeneration were also observed (Figure 3G). The dying hepatocytes shrank down to form a Councilman body (Figure 3H; indicated by the left arrow) and hepatocytes that became smaller with a condensed nucleus were noted (Figure 3H; indicated by the right arrow). The result suggested an involvement of apoptosis in the *K. pneumoniae*-evoked death of hepatocytes in mice with diabetes. Moreover, one of the *K. pneumoniae*-infected diabetic mice developed a large abscess in the right hepatic lobe (Figure 3I) and this was reminiscent of symptoms of human KLA disease. To quantify the degree of hepatic injury, all of the liver sections retrieved from the diabetic and naïve mice which had developed *K. pneumoniae*-caused liver infection were examined and graded using the Knodell scoring system [34]. Although there was no statistical significance, the average score of necroinflammatory lesions developed in the diabetic group (DI) was 1.8-fold higher than that in the *K. pneumoniae*-infected naïve mice (NI) (Figure 3J). 

**Table 1:** Incidence of developing extraintestinal *K. pneumoniae* infection in diabetic and naïve mice.

	Naïve mice (n=13)	Diabetic mice (n=10)	*P*-value^b^	Odds ratio	95% Confidence interval
Liver	4 (31%)	8 (80%)	0.036	9	1.285-63.025
Spleen	4 (31%)	5 (50%)	0.417	2.3	0.407-12.439
Kidneys	4 (31%)	5 (50%)	0.417	2.3	0.407-12.439
Blood	2 (15%)	5 (50%)	0.169	5.5	0.782-38.698
Incidence^a^	4 (31%)	8 (80%)	0.036	9	1.285-63.025

^a^ Incidence of extraintestinal *K. pneumoniae* infection was calculated as the percentage of mice that had positive culture of *K. pneumoniae* yielded in any of the extraintestinal organs tested. ^b^ Differences between the naïve and diabetic groups were determined by Fisher’s exact test. Statistical significance was determined based on a two-tailed *P* value ≤ 0.05.

### Inflammatory responses to *K. pneumoniae*-caused liver infection in diabetic and naïve mice

**Figure 4:**
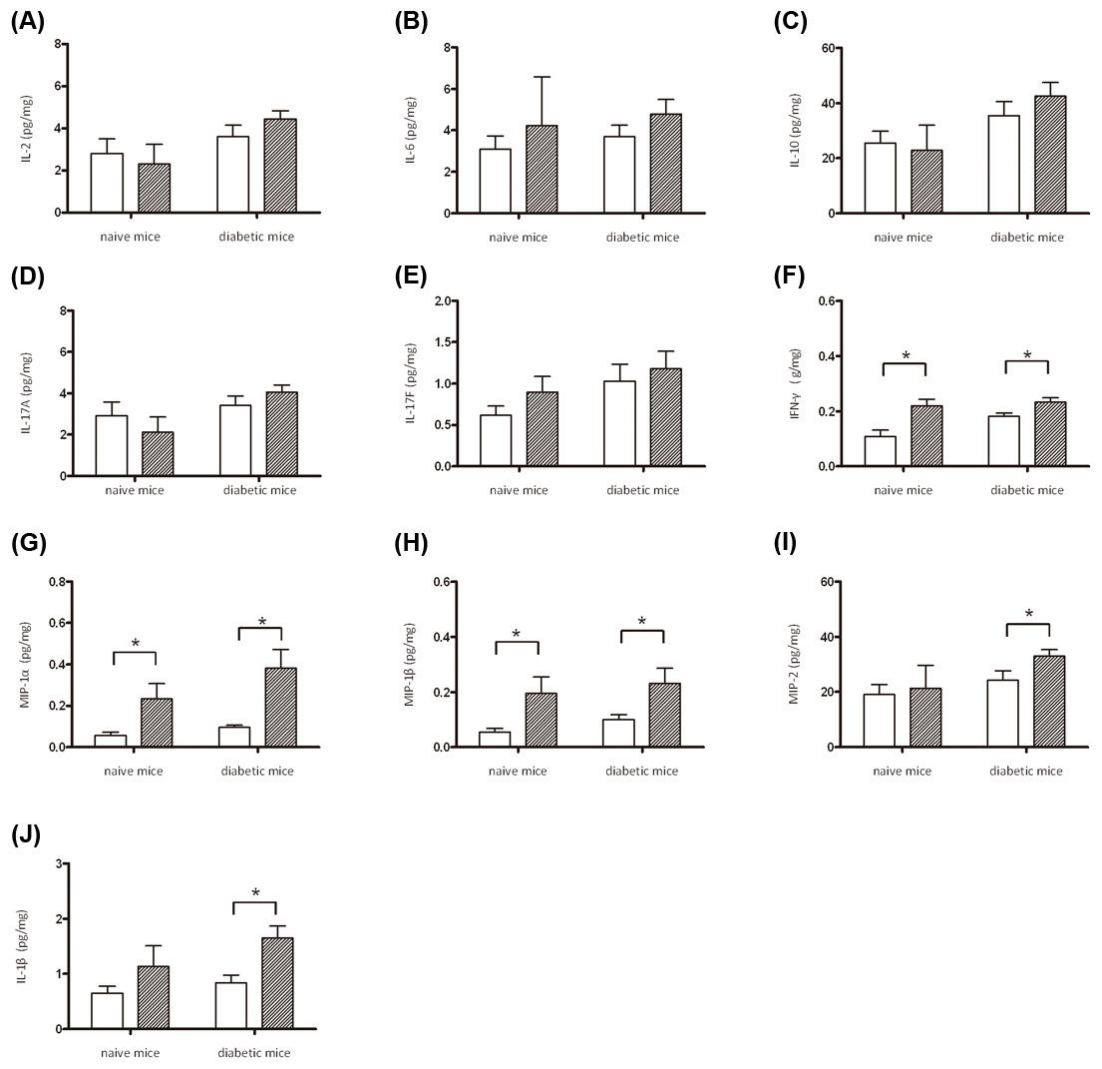
Production of cytokines and chemokines in hepatic responses to *K. pneumoniae* infection. Liver lysates were prepared from PBS-control naïve mice (n=3), PBS-control diabetic mice (n=3), and the *K. pneumoniae*-inoculated naive and diabetic mice which had developed an extraintestinal infection at 72 hpi (the sample size in naïve and diabetic groups is 4 and 8, respectively). Protein levels of IL-2 (A), IL-6 (B), IL-10 (C), IL-17A (D), and IL-17F (E), IFN-γ (F), MIP-1α (G), MIP1β (H), MIP-2 (I), and IL-1β (J) were determined by ELISA and normalized with total protein amounts. Data are expressed as the mean ± SEM. An asterisk (*) represents a significant increase in the *K*. *pneumoniae*-infected naïve or diabetic group (slash bar) in comparison with the corresponding control group (empty bar) by the Mann-Whitney U test (one-tailed; *P* < 0.05).

Excess proinflammatory responses can lead to uncontrolled tissue damage. Whether the severe liver injury caused by *K. pneumoniae* infection in the diabetic mice was attributed to a consequence of overwhelming inflammation was examined. As shown in Figure 4, level of cytokines and chemokines in the liver, including IL-2, IL-6, IL-10, IL-17A, IL-17F, IFN-γ, MIP-1α, MIP-1β, MIP-2, and IL-1β was determined in the diabetic and naïve mice which had developed *K. pneumoniae* infections by ELISA. Productions of IL-2 (Figure 4A), IL-6 (Figure 4B), IL-10 (Figure 4C), IL-17A (Figure 4D), and IL-17F (Figure 4E) were not significantly increased, probably because they were detected at 72 hpi, which represented a later stage for an acute *K. pneumoniae* infection. However, the production of IFN-γ (Figure 4F), which is the main Th1 cytokine that activates macrophages, was significantly evoked by *K. pneumoniae* infection at 72 hpi in both the diabetic and naïve mice and so were the chemokines released from the activated macrophages, MIP-1α (CCL3; Figure 4G) and MIP-1β (CCL4; Figure 4H). Nevertheless, upon *K. pneumoniae*-caused liver infection, stimulated productions of MIP-2 (CXCL-2) and IL-1β were both significantly stronger in the diabetic mice than in the naïve group (Figure 4I and Figure 4J). MIP-2 is a C-X-C chemokine that attracts neutrophils to the site of infection and IL-1β is a pivotal mediator of inflammatory response. Overall, the *K. pneumoniae*-evoked inflammation was enhanced by diabetes and that might lead to abscess formation and massive necrosis of liver parenchyma.

### Activation of IFN-γ/STAT/IRF-1 signaling in response to *K. pneumoniae* infection

**Figure 5:**
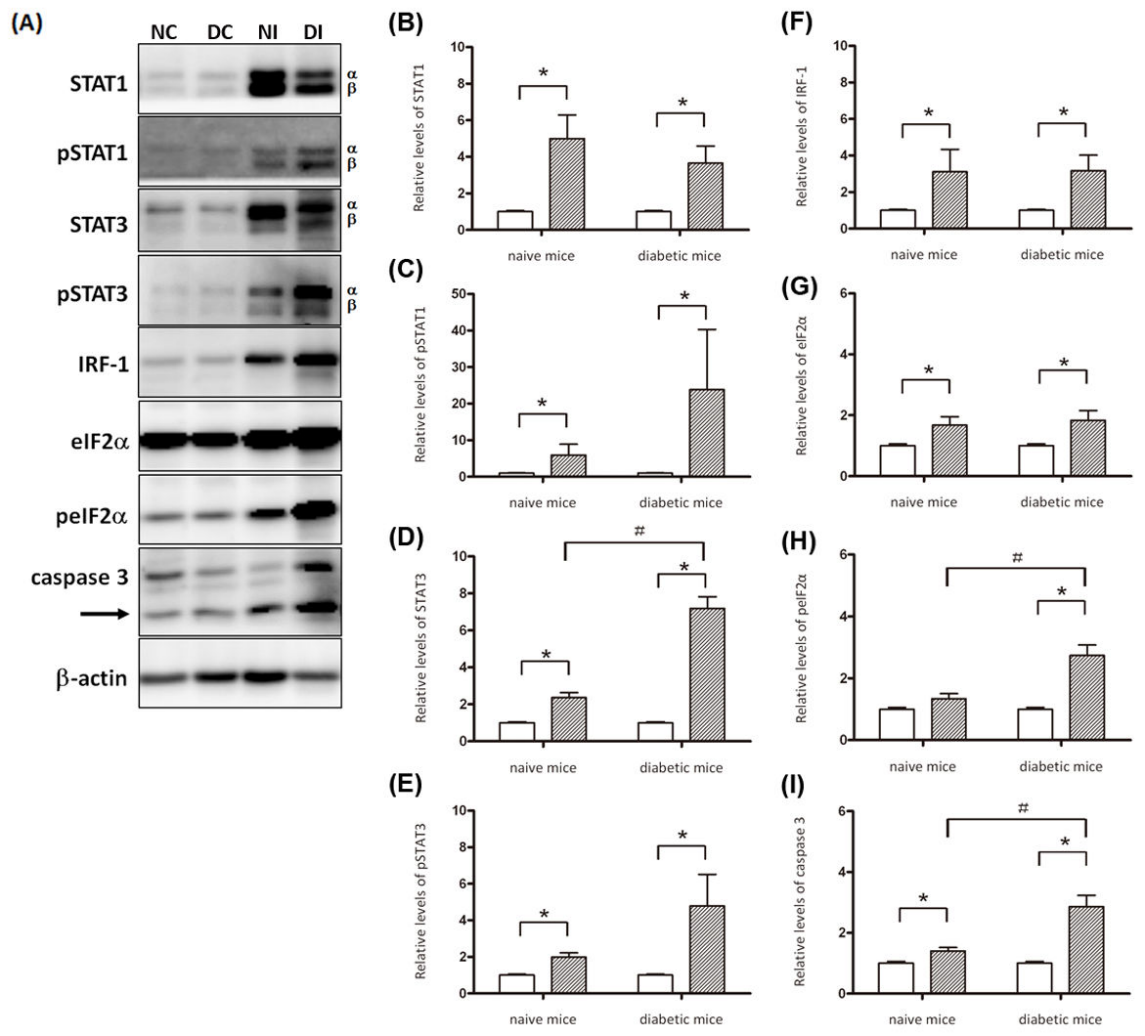
Activation of IFN-γ/STAT/IRF-1 signaling in hepatic responses to *K. pneumoniae* infection. Liver lysates were prepared from PBS-control naïve mice (n=3), PBS-control diabetic mice (n=3), and the *K. pneumoniae*-inoculated naive and diabetic mice which had developed an extraintestinal infection at 72 hpi (the sample size in naïve and diabetic groups is 4 and 8, respectively). Thirty micrograms of total proteins were subjected to Western blot analyses with specific antibodies. Western blotting analysis was repeated for three times by independent experiments. A representative result is shown in (A). Band intensity for each protein was determined by Densitometry calculation and normalized with β-actin. Data from three independent experiments for the expression level of (B) STAT1, (C) phospho-STAT1, (D) STAT3, (E) phospho-STAT3, (F) IRF-1, (G) eIF2α, (H) phospho-eIF2α, and (I) activated caspase 3 are shown as means ± SEM. Statistical analysis was performed by the Mann-Whitney U test (one-tailed). *P < 0.05 (one-tailed) represents a significant increase in the *K. pneumoniae*-infected naïve or diabetic group (slash bar) in comparison with the corresponding control group (empty bar). ^＃^ *P* < 0.05 (one-tailed) for significant difference between the naïve and diabetic mice which were *K. pneumoniae*-infected.

**Figure 6:**
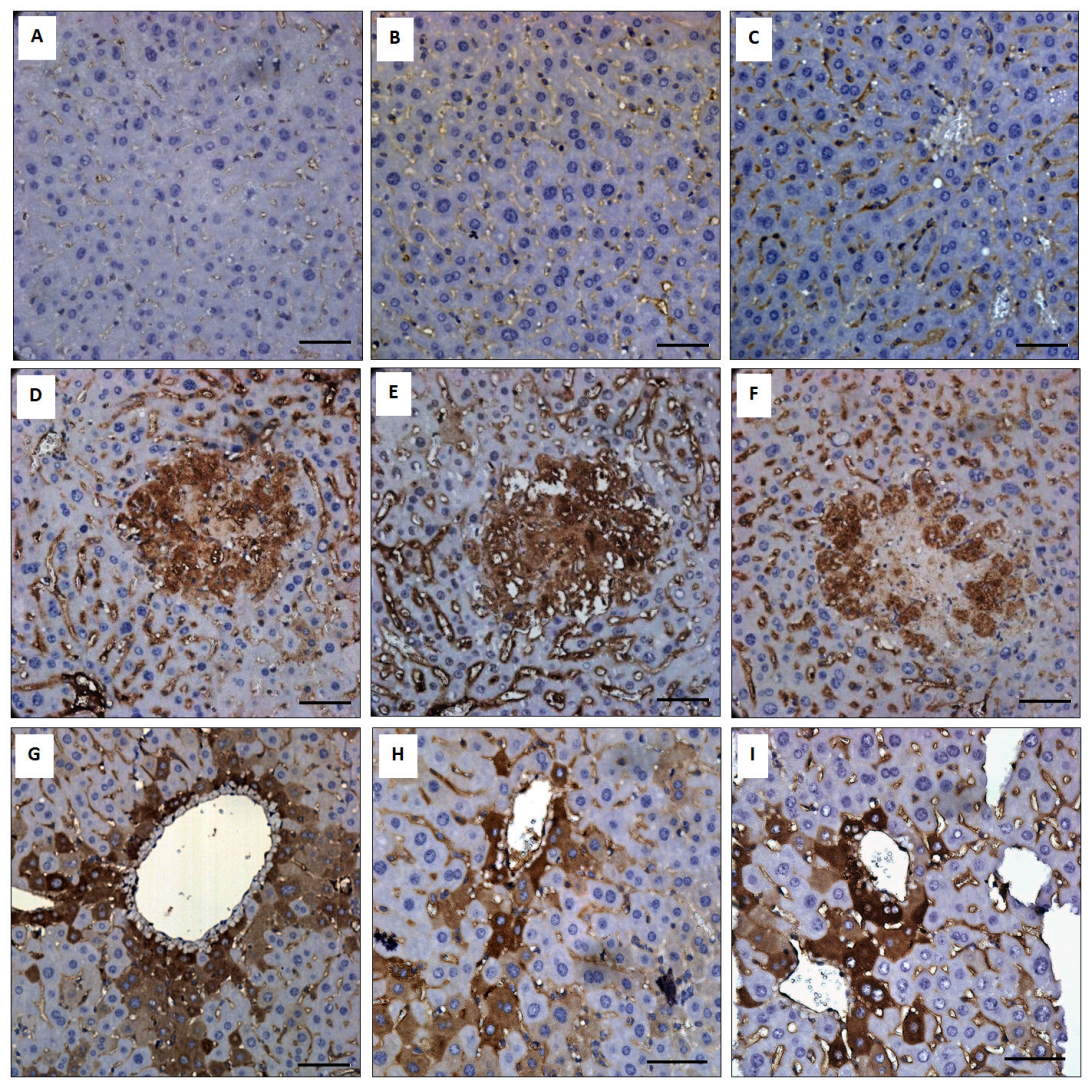
Immunohistochemistry analysis. Distribution of phospho-STAT1 (A, D, and G), phospho-STAT3 (B, E, and H), and IRF-1 (C, F, and I) are shown in the liver of the PBS-control (A, B, and C), *K. pneumoniae*-infected diabetic mice (D, E, and F), and *K. pneumoniae*-infected naïve mice (G, H, and I). Scale bar represents a distance of 50 μm.

IFN-γ was critical for the innate responses against pulmonary *K. pneumoniae* infections [28]. However, excessive production of IFN-γ impaired host resistance to bacteria, as a significant number of liver-specific IFN-γ transgenic mice died from enteric bacteremia [35]. As shown in Figure 4F, the production of hepatic IFN-γ was significantly increased in response to *K. pneumoniae* infection in both the diabetic and naïve mice. To address the role of IFN-γ in hepatic responses to *K. pneumoniae* infection, activation of IFN-γ/STAT/IRF-1 signaling was examined. As shown in Figure 5A-E, upon *K. pneumoniae* infections, the production of IFN-γ significantly elevated the level of expression and phosphorylation of STAT1 and STAT3 in the liver for both the diabetic and naïve mice. The formation of phospho-STAT1/STAT3 homo- and hetero-dimers transactivated the expression of downstream genes. The protein level of IRF-1 was elevated to 3-fold in response to *K. pneumoniae*-caused liver infection (Figure 5A and F). The activation of IFN-γ/STAT/IRF-1 pathway was confirmed by immunohistochemistry analyses. When compared with the PBS control (Figure 6A-C), positive signals for phospho-STAT1 (Figure 6D), phospho-STAT3 (Figure 6E), and IRF-1 (Figure 6F) were detected mainly within the foci of microabscess in the diabetic mice and were observed inside the liver parenchyma cells of *K. pneumoniae*-infected naïve mice (Figure 6G-H). The results demonstrated the activation of IFN-γ/STAT/IRF-1 pathway in hepatic response to *K. pneumoniae* infection. 

### Significant increases in the level of phospho-eIF2α and active caspase 3 in *K. pneumoniae*-infected diabetic mice

**Figure 7:**
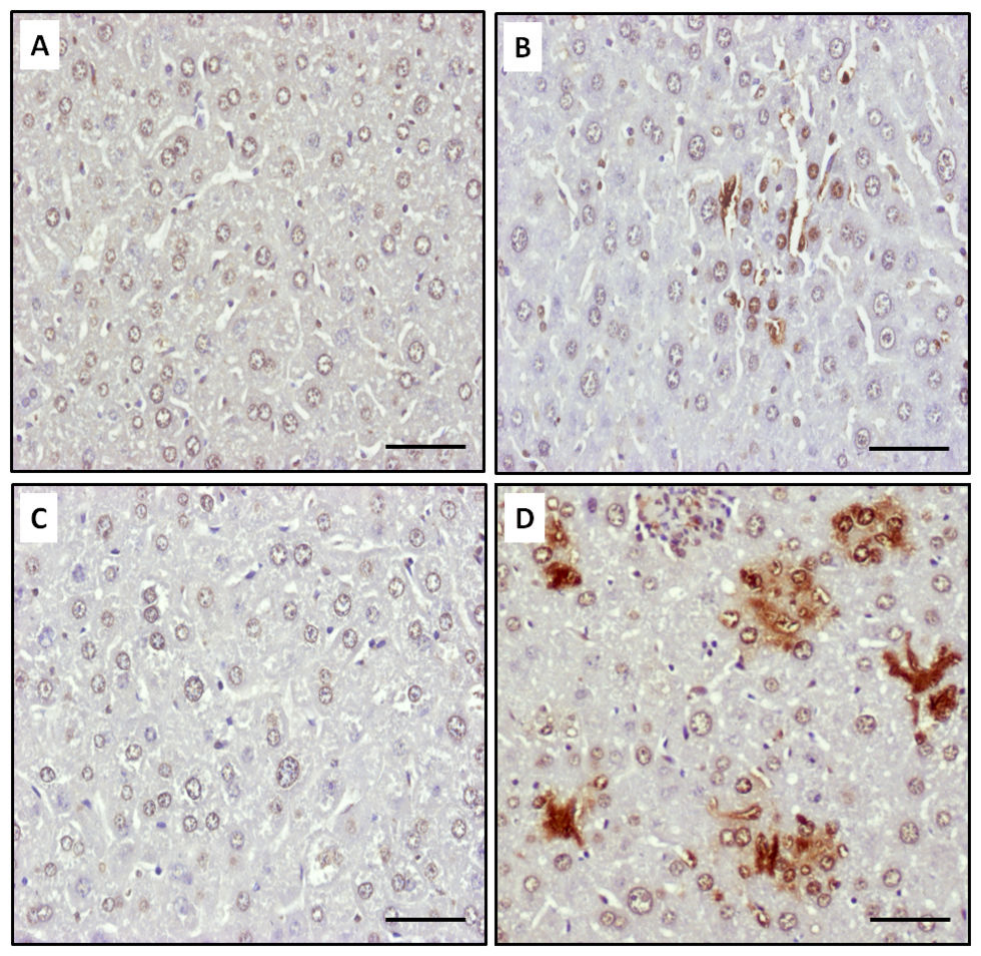
*K. pneumoniae*-induced hepatic apoptosis. Liver sections of PBS-control naïve mice (A), *K. pneumoniae*-infected naïve mice (B), PBS-control diabetic mice (C), and *K. pneumoniae*-infected diabetic mice (D) were subjected to TUNEL analysis. The nuclei of apoptotic cells have been stained brown with the TUNEL method. Scale bar represents a distance of 50 μm.

IFN-γ elicits apoptosis in a number of normal cells, including hepatocytes. Among the multiple pathways involved, ER stress has been shown to play a critical role in the signaling of IFN-γ induced apoptosis of primary hepatocytes [36]. Given that several apoptotic characteristics were noted in the *K. pneumoniae*-infected diabetic mice (Figure 3G and H), the involvement of ER stress was investigated. The level of phosphorylated eukaryotic initiation factor 2-alpha (peIF2α) (Figure 5A, G, and H), induced by PKR-like ER-localized eIF2α kinase (PERK) due to the ER protein load, was elevated exclusively in the diabetic mice upon *K. pneumoniae* infection. Moreover, protein level of p20 subunit of the activated caspase 3 that was proteolytically generated during apoptosis (Figure 5A; indicated by an arrow) was significantly increased in the *K. pneumoniae*-infected naïve and diabetic mice in comparison with the uninfected control (Figure 5I; * *P* < 0.05). To ascertain that the activation of caspase 3 was due to apoptosis, the liver sections retrieved from the naïve and diabetic mice which had *K. pneumoniae*-caused liver infection at 72 hpi were subjected to TUNEL assay. Apoptosis in a number of hepatic cells was induced by *K. pneumoniae* infection in both the diabetic and naïve mice (Figure 7). However, the significantly two-fold higher increase in the level of active caspase 3 in *K. pneumoniae*-infected diabetic mice in comparison with the naïve group (Figure 5I; ^#^ *P* < 0.05) suggested that *K. pneumoniae*-induced apoptosis was enhanced in mice with diabetes.

## Discussion

 To our knowledge, this is the first study that tracks the dissemination of *K. pneumoniae* in real time by using the bioluminescence imaging system. Overall, bacterial burdens of the *K. pneumoniae*-infected extraintestinal organs determined by the plating method correlated to the bioluminescence profiles obtained using the *in vivo* imaging method. The result suggests that the auto-bioluminescence-expressing plasmid pYC298 is relatively stable inside *K. pneumoniae* during infection. Although the resolution of the Xenogen IVIS system did not allow us to pinpoint the exact origin of signals, the autonomous bioluminescence imaging system established in this study provides a useful tool for real time monitoring of bacterial colonization and dissemination in deep organs of the same group of mice without the need for adding aldehyde substrate, which significantly minimizes the number of mice to be sacrificed.

 In Taiwan, approximately half of KLA patients have diabetes. In our mouse model, the incidence of developing an extraintestinal *K. pneumoniae* infection was significantly higher in mice with diabetes as compared with the age-matched naïve mice (Table 1; 80% *vs* 31%, *P*=0.036). Among the extraintestinal organs, liver was the primary site to be invaded by disseminating *K. pneumoniae* from the intestine (Table 1). In comparison to the naïve control, mice with diabetes were more prone to develop liver infection by *K. pneumoniae* (Table 1, odds ratio=9, 95% confident interval=1.285-63.025). This is consistent with epidemiological observation that diabetes is a major risk factor for the KLA disease. Intestinal colonization with *K. pneumoniae* is the first step of KLA development [32,33]. Previous studies demonstrated that type I diabetes patients were more likely to have mucosal alterations and increased intestinal permeability [37-39]. Our IVIS result (Figure 1C) suggested that small amounts of *K. pneumoniae* disseminating from the intestine were enough to initiate an extraintestinal infection. Thus, the vulnerability of diabetic mice to *K. pneumoniae*-caused liver infection may be attributed to increased intestinal permeability (impaired intestinal barrier function) of the diabetic host and/or enhanced intestinal colonization (reduced shedding) with *K. pneumoniae*.

 Once an extraintestinal infection was established, the proliferating capacity of *K. pneumoniae* in the diabetic mice was comparable to that in the naïve mice. As shown in Figure 2 E-H, bacterial burdens of the liver, spleen, kidneys and blood of the *K. pneumoniae*-infected diabetic mice resembled the average loads obtained from the naïve group. However, the severity of *K. pneumoniae*-evoked liver injury was obviously enhanced by diabetes. Extended proinflammatory response might have a role. In naïve mice, the production of proinflammatory cytokines induced by *K. pneumoniae* mostly returned towards basal levels at 72 hpi, whereas the expression of IL-1β and MIP-2 persisted in the diabetic mice. IL-1β is a pivotal inflammatory mediator, produced by PAMPs (Pathogen-associated molecular patterns)-activated cells of innate immune system, driving the host response to control infection or lead to tissue injuries [40]. Higher mRNA expression levels of IL-1β, MCP-1, and TNF-α have been demonstrated in the alveolar macrophages (AMs) of diabetic patients that failed to control infection in contrast to normal AMs [41]. Therefore, the lasting production of IL-1β, probably released by *K. pneumoniae*-activated Kupffer cells or neutrophils, might contribute to the enhancement of liver injury by diabetes (Figure 3). Besides, both IL-1β and MIP-2 are capable of attracting neutrophils to the site of infection. Although bacteria can be engulfed and killed by the recruited neutrophils, the release of cell-damaging intermediates such as free radicals elicits hepatic tissue damages [42]. The clearance of neutrophils from inflamed sites is required for limiting the inflammatory tissue damage and alleviating inflammation [43]. Therefore, the extended liver injury observed in the *K. pneumoniae*-infected diabetic mice might be attributed to the increased level of IL-1β and MIP-2 that induce tissue damage through recruitment of excessive neutrophils. 

 IFN-γ was essential for the innate response against pulmonary *K. pneumoniae* infection [28]. However, increased mortality of the liver-specific IFN-γ transgenic mice which died from enteric bacteremia [35] suggested that excessive IFN-γ production impaired host resistance to bacteria. Host defense against bacterial infection should be balanced between clearance of invading bacteria and avoidance of inflammatory tissue injury. In response to the liver-invaded *K. pneumoniae*, both the diabetic and naïve mice increased IFN-γ production (Figure 4A), probably from the activated innate lymphocytes that are abundant in the liver, including NK and natural killer T (NKT) cells. Recognition of IFN-γ by IFNGR1/2 expressed on hepatocytes, sinusoidal endothelial cells, and/or Kupffer cells activate STAT1 and STAT3 (Figure 5). The expression and phosphorylation level for both the α and β forms of STAT1 and STAT3 was significantly increased (Figure 5A) to mediate the *K. pneumoniae*-evoked IFN-γ signal. The subsequent formation of phospho-STAT1/STAT3 homo- or heterodimers, which translocated to the nucleus, activated the expression of IRF-1. In concanavalin A (ConA)-induced T cell hepatitis model, IFN-γ induced the expression of multiple chemokines (Mig, CCL-20, ENA-78, and IP-10) and adhesion molecules (ICAM-1 and VCAM-1), through the STAT-IRF1 dependent mechanism, which was critical for promoting infiltration of leukocytes into the liver and also for induction of apoptosis [16,17,44]. Transcriptome analysis of the *K. pneumoniae*-infected liver by DNA microarray platform (Agilent SurePrint G3 Mouse GE 8x60K Microarray) revealed that expression of IP-10, ICAM-1, and VCAM-1 was respectively up-regulated with 152-, 18-, and 25-fold change (unpublished data). Together, these findings suggested that the activation of IFN-γ/STAT/IRF-1 signaling was involved in the hepatic response to *K. pneumoniae*-caused infection. The IFN-γ responsive gene IRF-1 was responsible for subsequent activation of chemokines and adhesion molecules that recruited neutrophils to the site of infection and consequently aroused inflammatory tissue injury and abscess formation. 

The severity of hepatic tissue damage was enhanced in the *K. pneumoniae*-infected diabetic mice. In addition to inflammation-associated necrotic death, increased apoptosis of the liver parenchyma was noted in both the diabetic and naive mice, as evident by the result that p20 subunit of the activated caspase 3 was significantly elevated upon *K. pneumoniae* infection (Figure 5A and I). IFN-γ induces apoptosis of various cells, including hepatocytes. However, the mechanism is divergent and involves multiple pathways. Apoptosis of Hep3B and Chang-liver cells were triggered by IFN-γ activated phospho-STAT1 expression [45]. The STAT1-induced apoptosis involved the activation of caspase 2, 3 and 7 [46]. In ConA-induced hepatitis model, IFN-γ activated Fas-induced apoptosis pathway of hepatocyte [47]. Conversely, Fas-independent pathway was reported in IFN-γ induced apoptosis of primary murine hepatocytes that was mediated through IRF-1 [17]. In our KLA model, 3-fold elevation of phospho-eIF2α (Figure 5A, G and H) was revealed exclusively in the *K. pneumoniae*-infected diabetic mice. Phosphorylation of eIF2α suggests that protein translation is hold by inhibiting the formation of translation initiation complexes and is recognized as an indicator of ER stress. Accumulation of unfolded/misfolded proteins in ER lumen activates ER stress, also known as unfolded protein response (UPR). To restore normal function of the cell, expression of UPR-related genes attenuates protein translation and activates the production of chaperones involved in protein folding. However, the cells evolve towards apoptosis if the protein-folding error fails to be corrected and the disruption is prolonged [48]. The ER stress has been demonstrated to be involved in IFN-γ induced apoptosis in primary murine hepatocytes [17]. Although conditions related to obesity and diabetes activate ER stress in various tissues [49], the comparable levels of phospho-eIF2α exhibited in the PBS-control group of diabetic and naïve mice suggested that the diabetes-related ER stress was mainly provoked by *K. pneumoniae* infection (Figure 5H). Besides induction of ER stress, *K. pneumoniae* also caused 2-fold enhancement of hepatic apoptosis in mice with diabetes. These findings suggested that induction of ER stress by the liver-invading *K. pneumoniae*, probably through IFN-γ signaling, elevated the level of phospho-eIF2α to attenuate protein translation, and the failure to rescue ER stress led to augmented apoptosis in mice with diabetes.

Taken together, the activation of IFN-γ/STAT/IRF-1 signaling in the hepatic response to *K. pneumoniae* demonstrated by this work emphasizes the role of IFN-γ for mediating innate immunological responses, including macrophage activation, infiltrates of neutrophils, inflammatory tissue injury, and hepatic apoptosis. Prolonged production of IL-1β and MIP2, induction of ER stress, and increased apoptosis might contribute to *K. pneumoniae*-related hepatic damage in mice with diabetes. The relatedness of ER stress to apoptosis in hepatic response to *K. pneumoniae* infection in diabetic mice deserves a further study to elucidate.

## Materials and Methods

### Ethics statement

All animal experiments were performed in strict accordance with the recommendation in the Guide for the Care and Use of Laboratory Animals of the National Laboratory Animal Center (NLAC, Taiwan), and the protocol was approved by the Animal Experimental Center of Chung-Shan Medical University (Permit number: 1117). All surgery was performed under anesthesia, and all efforts were made to minimize suffering.

### Bacterial strains and plasmids

*K. pneumoniae* CG43, a K2 isolate from bacteremic liver abscess, exhibited strong virulence to BALB/c mice with an intraperitoneal LD_50_ of 10 CFU [50] and an oral LD_50_ of 2.6×10^6^ CFU [51]. For *in vivo* imaging studies, a bioluminescence-encoding plasmid, pYC298, was generated and introduced into *K. pneumonia* CG43 via electroporation. The 423-bp promoter region of *lon* gene was amplified by PCR with primers p186 (gAT ggT ACC gCg CTg CTT CgC gAC CTg) and p187 (AgA gAg CTC TgC gAg TCC TAA gTA TCT Cg), cloned into pDEW201-Tc^R^, which contained a copy of promoterless *luxCDABE* of *Photorhabdus luminescens* [29] as a reporter, and the resulting construct was named pYC298. The *lux*-expressing *K. pneumoniae* CG43-pYC298 exhibited strong bioluminescence during the regular culture with Luria-Bertani (LB) medium.

### Induction of diabetes in animals

As shown in our previous study [52], a multi-streptozotocin (STZ) injection method [53] was used to induce diabetes in mice. Briefly, male BALB/c mice were purchased from the National Laboratory Animal Center (NLAC, Taiwan) at the age of 6-wk-old and allowed to acclimatize in the animal house for one week before experiments. Mice with body weight 25-30 g were selected and divided randomly into two groups. One group received intraperitoneal injection of the pancreatic β-cell toxin streptozotocin (STZ; Sigma) for five days (55 mg/kg per day in 0.05 M sodium citrate buffer, pH 4.5) [53]; and the other group received sodium citrate buffer as the control. The serum glucose concentration and body weight of the mice were followed after the STZ injection. 

### Mouse infections

Mice with diabetes which was successfully induced by the multi-STZ-injection showing consistent serum glucose concentration ≥ 300 mg/dl were acclimatized in the animal house until the age of 15-week-old. To induce *K. pneumoniae* liver abscesses, the bacterial suspension containing 3 × 10^8^ CFU of *lux*-expressing *K. pneumoniae* CG43 was orally inoculated into groups of 15-wk-old diabetic mice and age-match naïve control. Twenty microliter of blood was retrieved from the retro-orbital sinus at indicative time points for bacterial enumeration. At 72 h post-inoculation (hpi), various mouse tissues were dissected from the control and infected mice and homogenized with PBS-0.2% Triton X-100. The 10-fold serial dilutes of tissue homogenates were plated onto LB agar supplemented with 10 μg/ml tetracycline for enumerating CFU of *K. pneumoniae*. 

### Liver histology and immunohistochemistry analysis

Livers retrieved from the control and *K. pneumoniae*-infected mice were fixed in 10% formalin, and processed for paraffin embedding. Liver sections were prepared with 5-10 μm thickness and stained with hematoxylin and eosin (H/E). For evaluation of hepatic injury, H/E sections were examined by experienced pathologists in a double-blinded fashion and the degree of hepatic injury for each section was graded using the Knodell necroinflammatory scoring system [34]. For detection of phospho-STAT1, phospho-STAT3, and IRF-1 in hepatic tissues, the paraffin-embedded sections were de-waxed with xylene and rehydrated in graded alcohol. The deparaffinized sections were then heated in 0.01 M citric buffer (pH 6.0) for 10 min for antigen-retrieval. Following treatment with 3% H_2_O_2_ in PBS at room temperature for 30 min, slides were washed, blocked with 5% normal goat serum in PBS and incubated respectively with rabbit monoclonal antibody against phospho-STAT1, phospho-STAT3, and IRF-1 (Cell signaling), at 4°C overnight. Positive signals were detected using NovoLink^TM^ Polymer Detection System (Leica Biosystem) according to the manufacturer’s protocol. Counterstaining was done with nuclear hematoxylin. For the terminal deoxynucleotidyl transferase-mediated dUTP nick-end labeling (TUNEL) assay, liver sections were stained using the In Situ Cell Death Detection kit (Roche Molecular Biochemicals), according to the manufacturer's instructions. 

### *In vivo* imaging of autonomous bioluminescence

The Xenogen IVIS Imaging System 200 Series was performed as previously described [54]. At 2, 5, 8, 24, 48, and 72 hpi after inoculation with 3 × 10^8^ CFU of *lux*-expressing *K. pneumonia* CG43, bacterial bioluminescence was directly detected, without the need of substrate addition, from the control and infected mice which were anaesthetized with isoflurane gas and maintained by administering 2-3% isoflurane through individual nose cones. Photons emissions from abdomen regions were detected by CCD camera and quantified using Living Image® software (Xenogen Corporation). *In vivo* luciferase activity was expressed as photons/second/cm^2^.

### Enzyme-linked immunosorbent assay (ELISA)

Expression level of cytokines and chemokines, including IL-1β, IL-2, IL-6, IL-10, IL-17A, IL-17F, IFN-γ, MIP-1α, MIP-1β, and MIP-2, in the liver lysates prepared from all experimental groups was measured using the standard ELISA kits (eBioscience) according to the manufacturer’s protocol and normalized with the quantity of total proteins.

### Western blot analysis

The liver retrieved from all experimental groups was homogenized with lysis buffer (8M urea, 50 mM DTT, 2% CHAPS, and complete protease and phosphatase inhibitors). The concentration of resulting suspensions was quantified with the 2-D Quant kit (GE Healthcare). Thirty microgram of liver proteins were resolved by 8% SDS-polyacrylamide gel and transferred onto a polyvinylidene difluoride (PVDF) membrane (Merck Millipore). After blocking with 2% of skim milk at room temperature for 1 h, the membrane was probed with each of the antibodies, including STAT1 (Cell signaling), STAT3 (Cell signaling), phospho-STAT1 (Cell signaling), phospho-STAT3 (Cell signaling), IRF-1 (Cell signaling), eIF2α (Cell signaling), phospho- eIF2α (Merck Millipore), and caspase 3 (Merck Millipore). After hybridization with HRP-conjugated secondary antibodies, the blots were developed with Immobilon^TM^ Western chemiluminescent HRP substrate (Merck Millipore) and visualized with ImageQuant^TM^ LAS 4000 mini biomolecular imager (GE Healthcare). The band intensities were quantified by UN-SCAN-IT gel 6.0 software. 

### Statistical analysis

Differences between the groups suggested were determined by Mann-Whitney U test or Fisher’s exact test. Statistical significance was determined based on a one-tailed or two-tailed *P* value < 0.05. All experiments were repeated a minimum of three times to ensure reproducibility.
